# In vivo dynamics of G-quadruplex DNA structures during liver regeneration in mice

**DOI:** 10.1038/s41598-026-51144-3

**Published:** 2026-04-28

**Authors:** Takumi Ishizuka, Kham Mo Aung, Baljinnyam Lkham-Erdene, Koichi Yano, Toshiki Kubota, Fidya, Shinichiro Shirouzu, Makoto Ikenoue, Kengo Kai, Phyu Synn Oo, Narantsog Choijookhuu, Yoshitaka Hishikawa

**Affiliations:** 1 Department of Anatomy, Histochemistry and Cell Biology, Faculty of Medicine, University of Miyazaki, 5200 Kihara, Kiyotake, Miyazaki, 889-1692 Japan; 2 Department of Thoracic Surgery, National Cancer Center of Mongolia, Nam Yan Ju street, Bayanzurkh District, Ulaanbaatar, 13370 Mongolia; 3 Department of Surgery, Faculty of Medicine, University of Miyazaki, Miyazaki, 889-1692 Japan; 4 Department of Oral and Maxillofacial Surgery, Faculty of Medicine, University of Miyazaki, 5200 Kihara, Kiyotake, Miyazaki, 889-1692 Japan; 5 Department of Oral Biology, Faculty of Dentistry, Universitas Brawijaya, Malang, Jawa Timur 65145 Indonesia; 6 Present Address: Department of Pathology and Forensic Medicine, School of Biomedicine, Mongolian National University of Medical Sciences, Zorig street, Ulaanbaatar, 14120 Mongolia; 7 Frontier Science Research Center, University of Miyazaki, 5200 Kihara, Kiyotake, Miyazaki, 889-1692 Japan

**Keywords:** G-quadruplex, Liver regeneration, Partial hepatectomy, HMGB2, Biochemistry, Cell biology, Molecular biology

## Abstract

Guanine-rich DNA sequences can form four-stranded structures known as G-quadruplexes (G4s), which play important roles in cellular processes. However, direct observation of G4 dynamics in tissues remains challenging. Here, we report the in vivo dynamics of G4s during mouse liver regeneration after partial hepatectomy (PHx) using a G4-specific antibody. G4-positive hepatocytes gradually increased after PHx, peaking at 36 h, and then decreased, with signals predominantly in nuclei of cells in the peripheral region between the periportal and midzonal regions. Notably, double immunofluorescence analysis of cell cycle markers revealed that G4-positive cells colocalize with EdU- and Cyclin A2-positive cells, suggesting that G4 structures are formed during the S to G2 phases of the cell cycle. In mice deficient in high-mobility group box 2 (HMGB2), a candidate G4-interacting protein, G4-positive cells were significantly reduced compared with wild-type controls, although the temporal peak was preserved. Spatial distribution of G4-positive hepatocytes was also altered in *Hmgb2*^*−/−*^ mice. This study provides the first evidence that G4-binding protein knockout mice can be used to assess the effects of these proteins on DNA G4 formation in vivo.

**Supplementary Information:**

The online version contains supplementary material available at 10.1038/s41598-026-51144-3.

## Introduction

Genomic DNA, known for its right-handed double-helical structure, can also adopt several secondary structures^1,2^. A prominent example is the DNA G-quadruplex (G4), which specifically forms in regions of the genome that are rich in guanine^3,4^. G4s are four-stranded nucleic acid structures consisting of stacked planar structures made of four guanines (known as a G-quartet) that associate through Hoogsteen hydrogen bonding, and the structure is stabilized by a central monovalent cation^5^. Pioneering studies have defined the G4 consensus motif as G_3−5_N_1−7_G_3−5_N_1−7_G_3−5_N_1−7_G_3−5_, where N represents any nucleotide. Computational algorithms based on this consensus have predicted that the human genome contains 376,000 sequences with the potential to form G4s^6^. Studies using recently established high-throughput and genome-wide methods for profiling G4s with high resolution have revealed that the human genome contains more than 716,000 potential G4-forming sequences^7^.

Studies have shown that G4s are present in human cells, and their formation in live cells is dynamic^2,8^. G4s have attracted significant attention as novel molecular targets in the treatment of various diseases, and extensive research has thus focused on this topic^1,9–15^. Also, G4s play significant roles in transcription and replication, and they have been associated with genome instability and transcriptional regulation^4,16–37^. G4 chromatin immunoprecipitation sequencing (G4 ChIP-seq) studies revealed that endogenous G4s are enriched in open chromatin regions and promoters of highly expressed cancer genes^38,39^. Increasing evidence indicates that G4s are formed in living cells and play important biological roles. However, visualizing the dynamics of G4 structures in vivo is challenging. In particular, although the role and functions of G4s in vitro and in cells have been extensively studied using chemical methods, they have not been studied in vivo.

High-mobility group box 2 (HMGB2), a non-histone, chromatin-associated protein that plays a pivotal role in DNA architecture, transcriptional regulation, and genome stability^40,41^, was recently identified as a candidate protein associated with G4s in living cells. This discovery was made using the co-binding–mediated protein profiling strategy, which employs G4-specific small-molecule photocrosslinking probes^42^. Through this approach, HMGB2 was found to localize in close proximity to G4s without directly interacting with them. However, the in vivo dynamics of G4-associated proteins such as HMGB2 and of G4 structures remain largely unexplored. Importantly, to elucidate the biological functions of G4s, including their in vivo dynamics, it is critical to examine tissues and organs in which individual cells are spatially organized.

The liver consists of a variety of cell types, including hepatocytes, biliary epithelial cells, sinusoidal endothelial cells, stellate (Ito) cells, and Kupffer cells. Hepatocytes, which are responsible for the majority of the liver’s metabolic and synthetic activities, comprise approximately 80% of liver weight and approximately 70% of the total liver cell population^43^. Notably, the liver is a distinctive organ that exhibits a remarkable ability to regenerate^44^. Partial hepatectomy (PHx) has been extensively utilized as a preferred model for studying liver regeneration^45–51^. After surgical removal of 70% of a rodent’s liver, cell division and proliferation are automatically initiated in the remaining liver tissue, resulting in rapid regeneration until the organ reaches its original volume and weight, at which time the response self-terminates. In rodents, most of the liver mass is restored within 7 − 8 days after PHx. Given that resectioning of liver lobes does not damage the residual tissue, PHx serves as a clean experimental model ideally suited for investigating cell cycle dynamics in vivo.

In the present study, we investigated the dynamics of G4s during liver regeneration using a 70% PHx mouse model. Using a G4-specific antibody, we first evaluated changes in G4 formation during liver regeneration by immunohistochemistry analysis of mouse liver samples collected at defined time periods after 70% PHx. The spatiotemporal formation of G4s during hepatocyte proliferation was then demonstrated by analysis of various cell cycle–specific markers. To further characterize the spatiotemporal formation of G4s, we investigated G4 formation during liver regeneration using a 70% PHx model in wild-type (WT) and HMGB2 knock-out (*Hmgb2*^*−/−*^) mice, as HMGB2 has been identified as a candidate G4-binding protein.

## Results

### Spatiotemporal formation of G4 in hepatocytes during liver regeneration

**Fig. 1:**
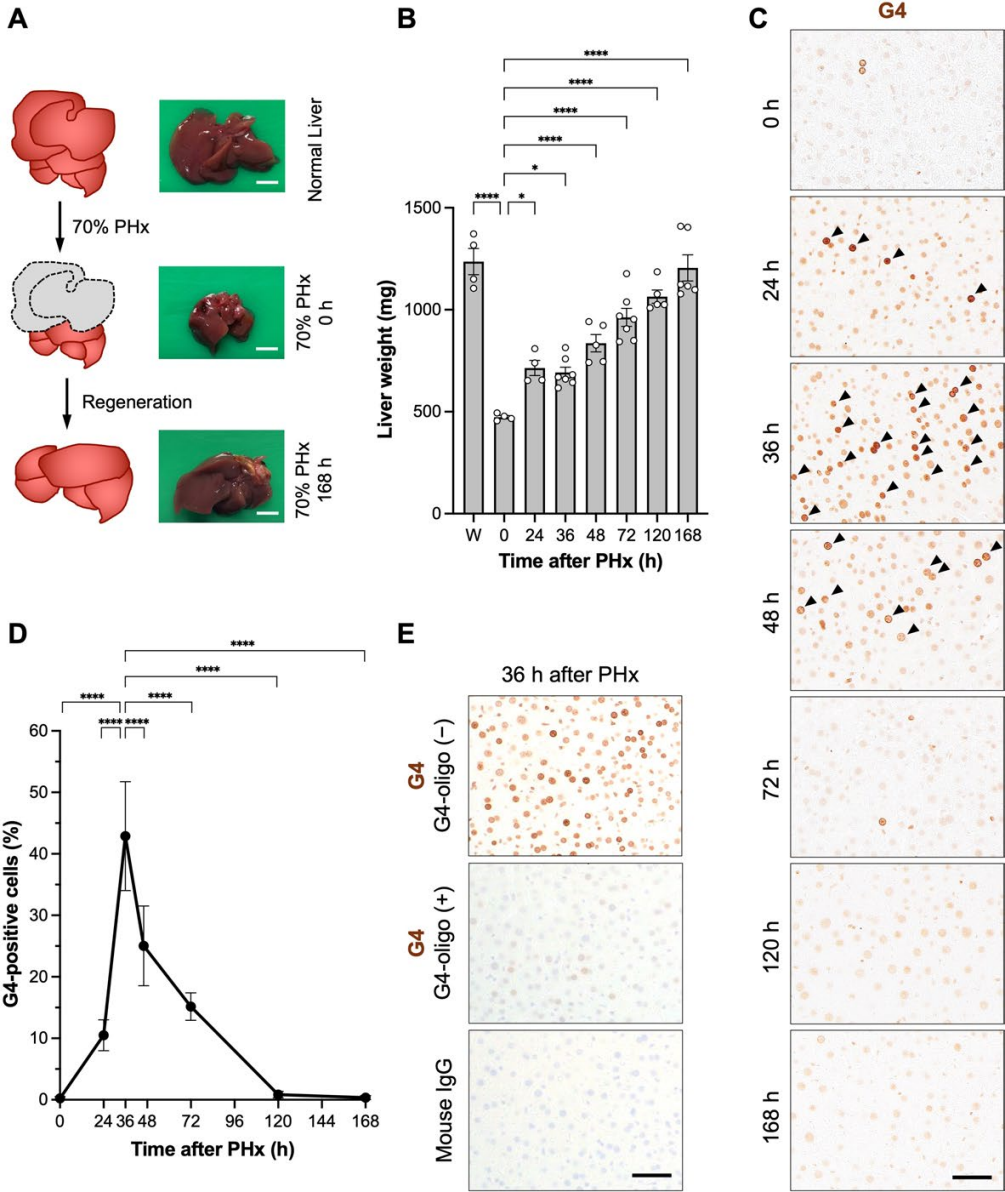
G4 formation in mouse liver during regeneration. (**A**) Schematic representation of liver regeneration. After 70% PHx, the original liver mass was recovered within 7 days. A normal mouse liver after 70% PHx (0 h) and regenerated liver after 70% PHx (168 h) are shown at the bottom. The median and left lobes were surgically removed during 70% PHx. Scale bars represent 5 mm. (**B**) Change in liver weight during regeneration. Data represent the mean ± SEM for 4–7 mice. W, whole liver before PHx. (**C**) Detection of G4s during liver regeneration by immunohistochemistry analysis. Time after PHx is shown on the left. Arrowheads indicate G4-positive hepatocytes detected during liver regeneration. (**D**) Post-PHx percentage of G4-positive hepatocytes in the liver. Data represent the mean ± SEM from 5–6 mice per group. (**E**) Neutralization assays to evaluate G4 antibody immunoreactivity in liver tissues. Liver tissues at 36 h post-PHx were treated without (upper panel) or with (middle panel) an excess of G4 oligonucleotides that were allowed to form G4 structures prior to incubation with the anti-G4 antibody. Mouse IgG was applied instead of the anti-G4 antibody (bottom panel). In (**B**) and (**D**), *p* values for differences between each time point and day 0 were calculated using one-way ANOVA followed by Dunnett’s test (*p < 0.05, **p < 0.01, ***p < 0.001). In (**C**), (**E**), and (**F**), nuclei were counterstained with hematoxylin. Scale bar, 50 μm.

To investigate the formation of G4 during liver regeneration in mice, we performed 70% PHx in WT mice and collected remnant liver tissues at various time points. Liver mass increased following 70% PHx, with the original mass recovered by 7 days (168 h), as previously reported^52^ (Fig. 1A and B). The formation of G4 structures during liver regeneration was examined using a G4-specific antibody that has been employed for immunohistochemical analyses^53–57^. Interestingly, the number of G4-positive cells began to increase at 24 h post-PHx, peaking at 36 h before decreasing and eventually disappearing by 168 h (Fig. 1C). The counting results of G4-positive cells also confirmed that a peak was observed in liver samples at 36 h post-PHx, with 42.9 ± 8.9% of all hepatocytes analyzed determined to be G4 positive (Fig. 1D). Immunocytochemistry analysis of synchronized cell populations revealed that the rate of G4 formation was lowest during the G_0_/G_1_ phase, a quiescent stage of the cell cycle in which DNA replication does not occur. By contrast, the number of G4-positive cells increased markedly during the S phase, showing approximately 6.3 times more G4-positive cells than in the G_0_/G_1_ phase (Supplementary Fig. S1A and B). These supplementary in vitro analyses demonstrated that the specificity of the antibody used in this study was consistent with that reported in previous studies^58^, thereby validating its suitability for the present study.

The suitability and specificity of the G4-specific antibody were also evaluated using a neutralization assay. The anti-G4 antibody was preincubated with G4-forming oligonucleotides, and then immunostaining was carried out on liver tissue sections. When the antibody was preincubated with G4-forming oligonucleotides prior to application to liver tissue sections for immunostaining, no detectable G4 signals were observed (Fig. 1E). To determine whether the results of this immunohistochemical analysis were derived from DNA G4s, enzymatic treatment (DNase) was performed prior to immunostaining. No signals were observed following DNase treatment, whereas RNase treatment had no effect on G4 signals (Supplementary Fig. S1C). These results indicate that the signals originated from DNA G4s.

**Fig. 2:**
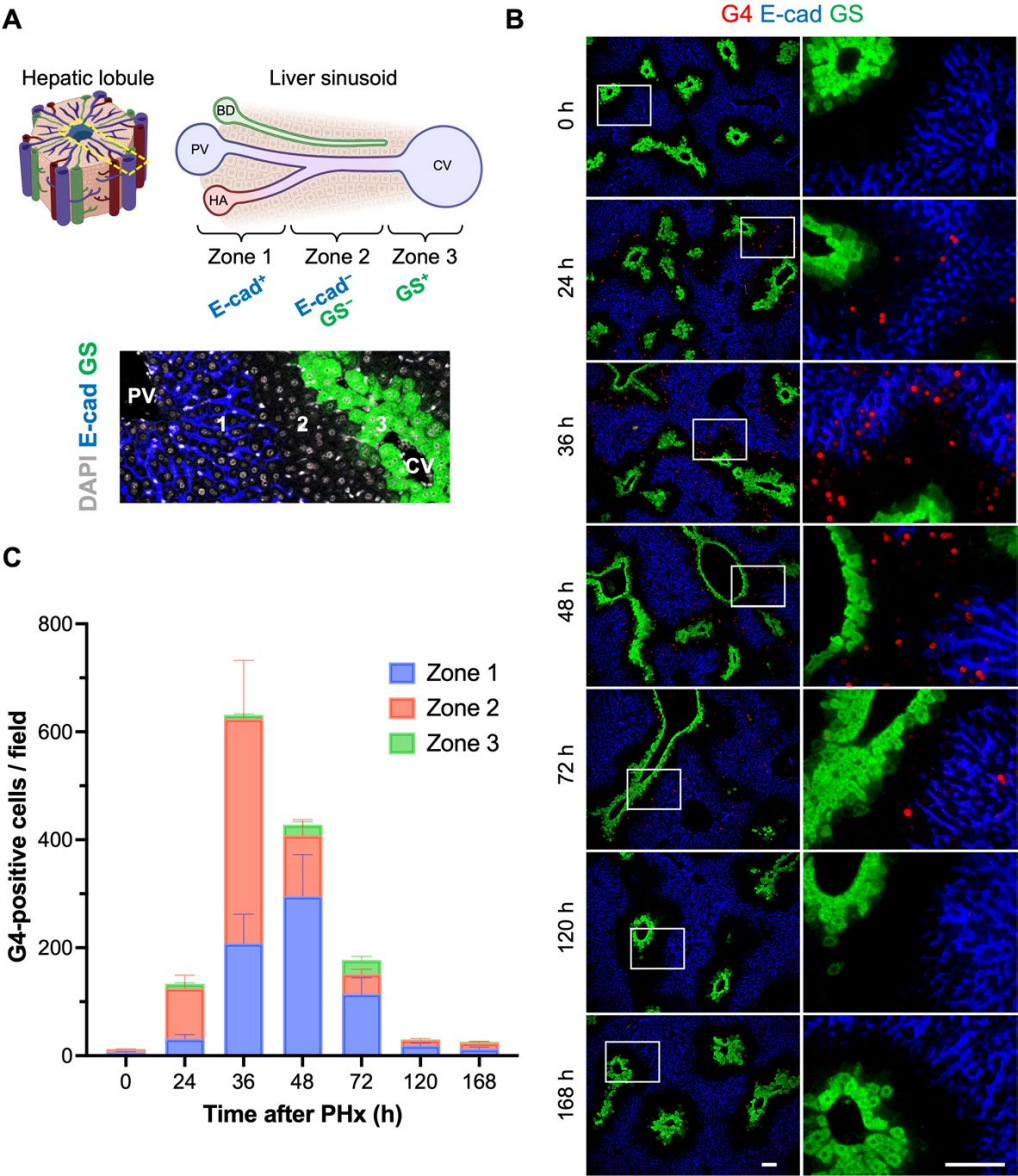
G4 structures form in zone 2 hepatocytes during liver regeneration. (**A**) Schematic representation of the three hepatic zones from the periportal to the pericentral region. Lower panel shows results of immunostaining for glutamine synthetase (GS) and E-cadherin (E-cad). Zones 1, 2, and 3 correspond to the E-cad⁺ (zone 1), E-cad⁻/GS⁻ (zone 2), and GS⁺ (zone 3) regions, respectively. In the liver sinusoid, PV, CV, BD, and HA indicate the portal vein, central vein, bile duct, and hepatic artery, respectively. (**B**) Immunofluorescence analysis of G4 (red), E-cad (blue), and GS (green) expression in the liver of mice post-PHx. Time after PHx is shown on the left. Boxed areas are enlarged in the right panel. Scale bar, 100 μm. (**C**) Quantification of G4-positive hepatocytes in each zone of the liver of mice post-PHx. The number of G4-positive cells was determined among 2,000 hepatocytes. Data represent the mean ± SEM from 5–6 mice per group.

We further analyzed the spatiotemporal formation of G4 during liver regeneration. It is important to note that hepatocyte populations are not homogeneous; rather, their functions are spatially organized based on location within the hepatic lobule^59^. Structurally, each lobule is centered on a central vein and surrounded by portal triads composed of a branch of the portal vein, hepatic artery, and bile duct. Zone 1 is located near the portal triad, whereas zone 3 surrounds the central vein, and zone 2 is situated between these areas. Several molecular markers have been established for distinguishing the three distinct zones of the liver lobule^60^ (Fig. 2A). Immunostaining for G4, E-cadherin (E-cad, for zone 1), and glutamine synthetase (GS, for zone 3) revealed that at 36 h post-PHx, when G4 signals reached their peak, the signals were predominantly localized in zone 2, particularly in the marginal zone adjacent to zone 1 (Fig. 2B and Supplementary Fig. S2). Quantification of G4-positive hepatocytes at seven time points revealed the percentage of G4s formed in each zone during liver regeneration (Fig. 2C). Interestingly, the number of G4-positive hepatocytes was significantly lower in zone 3 during liver regeneration compared with zones 1 and 2. Furthermore, the percentage of G4-positive hepatocytes in zone 2 was 2 times higher than that in zone 1 at 36 h post-PHx. By 48 h post-PHx, the initial enrichment in G4 formation in zone 2 had expanded into zone 1, resulting in a reversal of the zonal distribution. After 48 h post-PHx, an increase in the number of G4-positive hepatocytes in zone 1 was observed. The number of G4-positive hepatocytes in zone 1 was approximately 2.5 times greater than that in zone 2. The number of G4 signals in zone 2 was significantly reduced at 72 h post-PHx, whereas the number of G4 signals in zone 1 became higher than that in zone 2. In addition, several G4-positive hepatocytes also appeared in zone 3. These findings highlight the dynamic and region-specific modulation of G4 structures throughout the liver regeneration process. Collective data from continuous recording of all hepatocytes during liver regeneration suggest that the rate of G4 formation in hepatocytes is highest in zone 2, relatively modest in zone 1, and lowest in zone 3.

### Colocalization of G4 and cell cycle markers during liver regeneration

To investigate whether G4 formation is affected by cell-cycle status, we performed immunofluorescence analyses using specific cell cycle markers. Colocalization of G4 and Ki-67, which is expressed from the G_1_ to M phase and absent in the G_0_ phase, was analyzed to examine G4 formation during liver regeneration (Supplementary Fig. S3A). Consistent with previous studies^61,62^, we also confirmed that hepatocytes actively entered the cell cycle at 48 h post-PHx, whereas the number of Ki-67-positive cells was reduced at 72 h post-PHx. By contrast, G4 signals were observed at 36 and 48 h post-PHx, with a peak at 36 h. G4 signals were detected 12 h earlier than Ki-67 signals (Supplementary Fig. S3C).

We then evaluated G4 formation in the context of the expression of proteins that control cell cycle progression. Cyclin D1 functions during the G_1_ phase of the cell cycle, in which the cell prepares to initiate DNA synthesis, and it plays a role in regulating the G_1_/S transition^63^. No colocalization was observed with the G_1_/S phase marker cyclin D1 (Supplementary Fig. S3B and D). To further confirm these results, we investigated the expression of PCNA as an additional marker. PCNA is a marker of the late G_1_/S phase and an essential factor in DNA replication^64^. Fluorescence imaging of G4 and PCNA revealed partial colocalization (< 10% of hepatocytes), which peaked at 36 h post-PHx (Supplementary Fig. S4A and C).

**Fig. 3:**
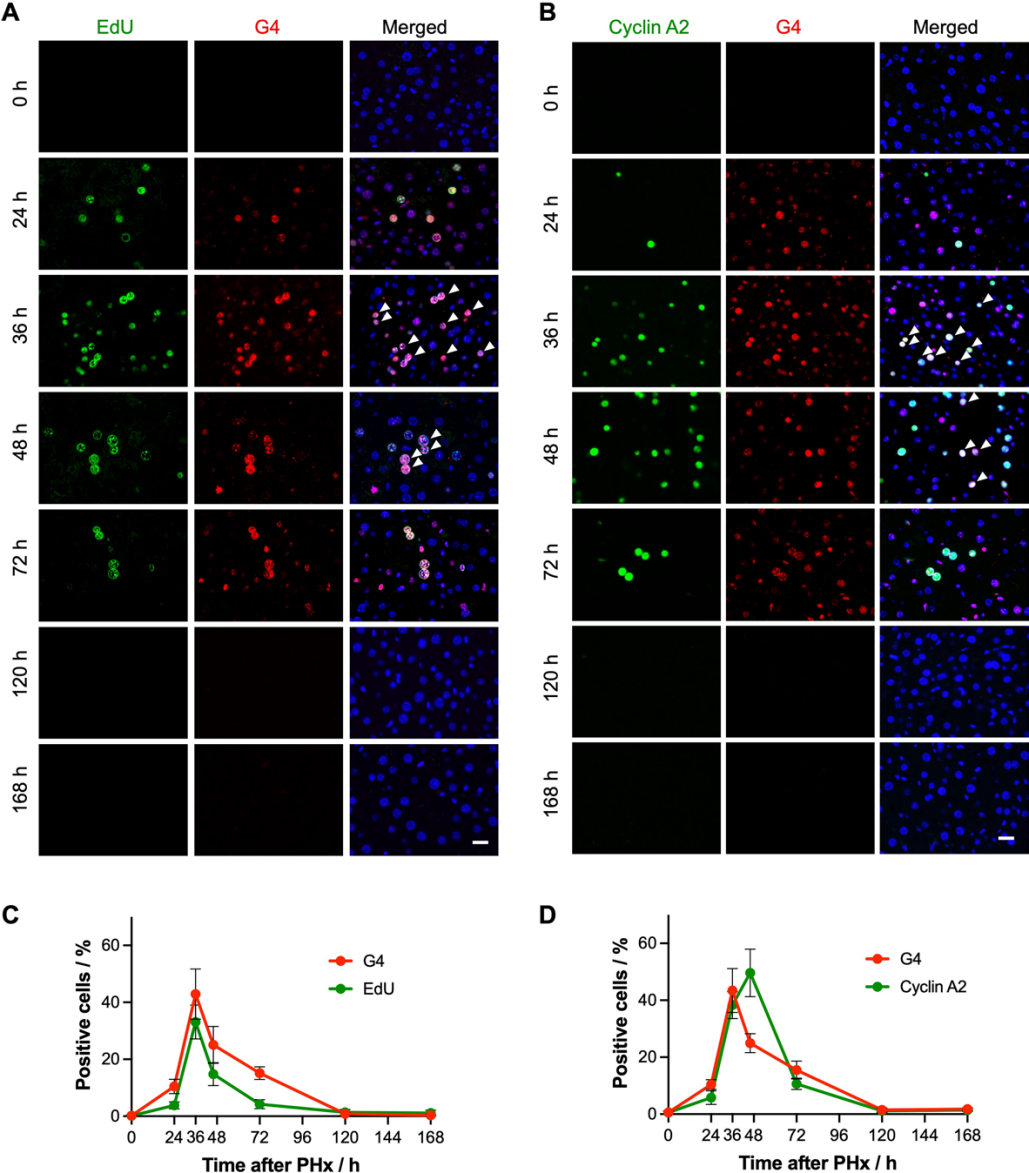
Colocalization of G4 with EdU and cyclin A2 during liver regeneration. (**A**) Immunofluorescence analysis of EdU (green) and G4 (red) expression in the liver of mice post-PHx. (**B**) Immunofluorescence analysis of cyclin A2 (green) and G4 (red) expression in the liver of mice post-PHx. Scale bar, 20 μm. Arrowheads indicate double-positive hepatocytes observed during liver regeneration. Numbers of EdU-positive cells (**C**) and cyclin A2-positive cells (**D**) in the liver of mice post-PHx. Data represent the mean ± SEM from 4–7 mice per group.

**Fig. 4:**
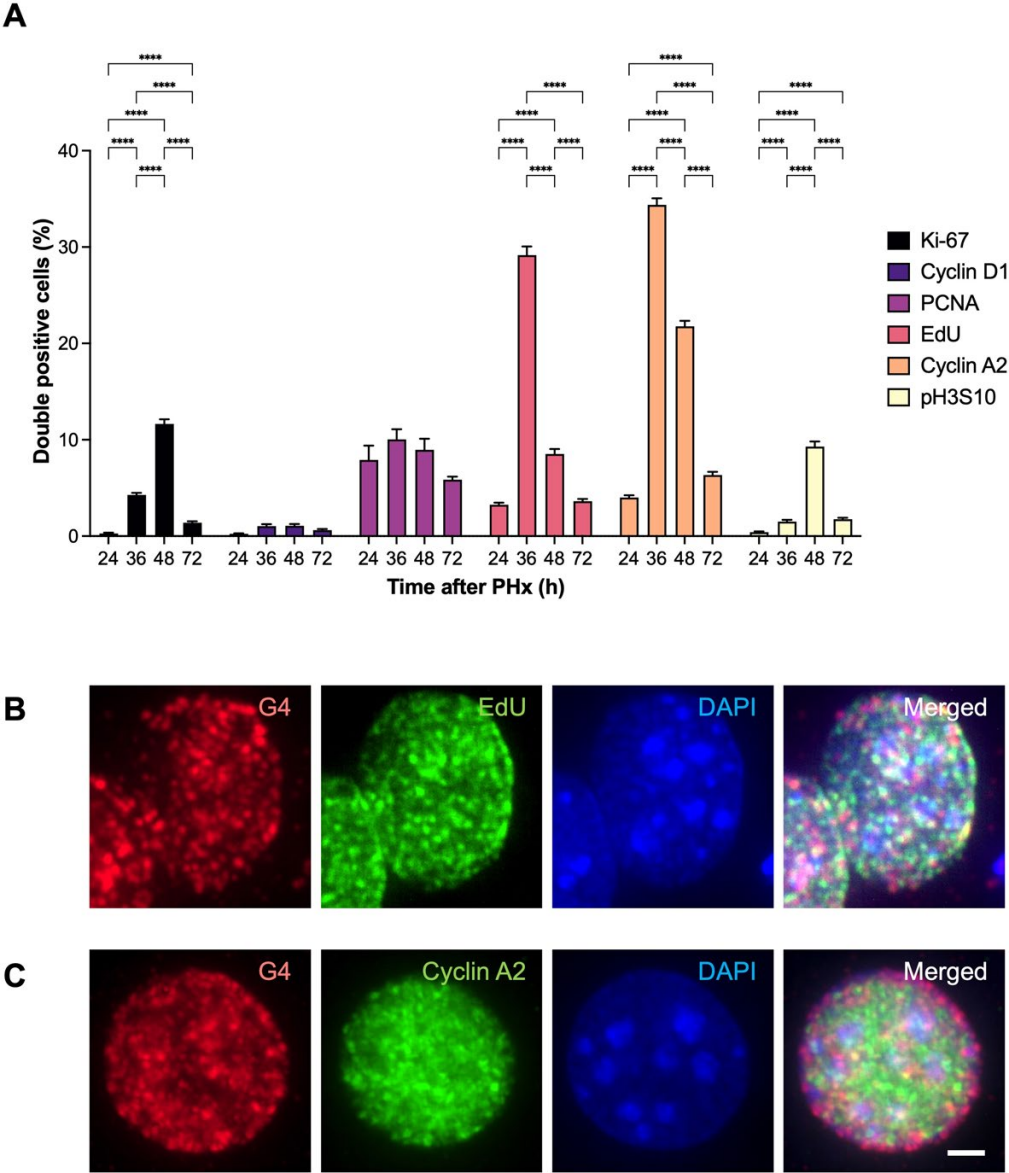
Evaluation of hepatocytes double positive for G4 and various cell cycle markers during liver regeneration. (**A**) Quantification of cells double positive for G4 and each cell cycle marker at 24, 36, 48, and 72 h post-PHx. Statistical comparisons of data sets were performed using a mixed-effects model followed by Tukey’s multiple comparisons test (*p < 0.05, **p < 0.01, ***p < 0.001). Super-resolution imaging of nuclei showing colocalization of G4 with EdU (**B**) or cyclin A2 (**C**) in mouse liver at 36 h post-PHx. Scale bar, 2 μm.

To further characterize the relationship between G4 formation and cell cycle progression, we examined the expression of additional markers associated with DNA synthesis and mitosis. Staining for EdU to identify hepatocytes in S-phase^65^ revealed that both G4 and EdU signals peaked at 36 h post-PHx, with colocalization observed in 29% of hepatocytes (Fig. 3A and C). G4 formation was further examined with regard to the expression of cyclin A2, which also plays an important role in progression of the cell cycle. Cyclin A2 is expressed during the S phase, G_2_ phase, and early mitosis, and it is thus commonly used as a marker of proliferating cells due to its function as an essential regulatory factor controlling a large portion of the cell cycle^66–68^. G4 and cyclin A2 signals colocalized in 34% and 22% of hepatocytes at 36 and 48 h post-PHx, respectively (Fig. 3B and D). Finally, we analyzed the colocalization of G4 with phosphorylated H3S10 (pH3S10) to investigate G4 formation during the M phase. At 48 h post-PHx, only a small number of pH3S10-positive hepatocytes exhibited co-staining with G4, whereas the majority of hepatocytes showed no colocalization, suggesting that G4 is not involved in M phase (Supplementary Fig. S4B and D). The proportions of hepatocytes showing colocalization of G4 and various cell cycle markers are summarized in Fig. 4A. Notable differences were observed for EdU and cyclin A2, with 29.1 ± 0.9% and 34.4 ± 0.7% of hepatocytes, respectively, showing colocalization at 36 h post-PHx. Additionally, colocalization of G4 and cyclin A2 signals was observed in 21.8 ± 0.6% of hepatocytes at 48 h post-PHx. Collectively, these data indicate that over the course of liver regeneration, G4 formation increases as hepatocytes progress toward the S phase and persists into the post-S phase, including the G2 phase.

The subcellular localization of G4 was also investigated using super-resolution microscopy, which provides a resolution of approximately 120 nm for visualizing target organelles. Interestingly, dot-like G4 signals were observed in the nuclei of hepatocytes, where some of these signals colocalized with EdU (Fig. 4B). By contrast, G4 signals in hepatocyte nuclei that colocalized with cyclin A2 appeared diffuse (Fig. 4C). These findings suggest that G4 exhibits distinct behaviors depending on the specific phase of the cell cycle. Taken together, these results suggest that DNA G4s are associated not only with cell cycle progression during DNA synthesis but also with post-synthetic phases during post-PHx liver regeneration.

### G4 formation is reduced during liver regeneration in HMGB2-KO mice

Given that DNA G4 formation during liver regeneration showed clear cell cycle–dependent and zonation-specific patterns, we hypothesized that specific nuclear factors contribute to the stabilization or resolution of G4 structures in hepatocytes. Although several nuclear proteins have been identified as potentially binding G4^42,69,70^, their physiological relevance in the regenerating liver remains unclear. In particular, proteins that recognize or remodel G4 structures may regulate hepatocyte proliferation through direct modulation of the conformation of DNA. To explore this possibility, we examined HMGB2, a chromatin-associated protein with known affinity for distorted DNA structures^71,72^.

**Fig. 5:**
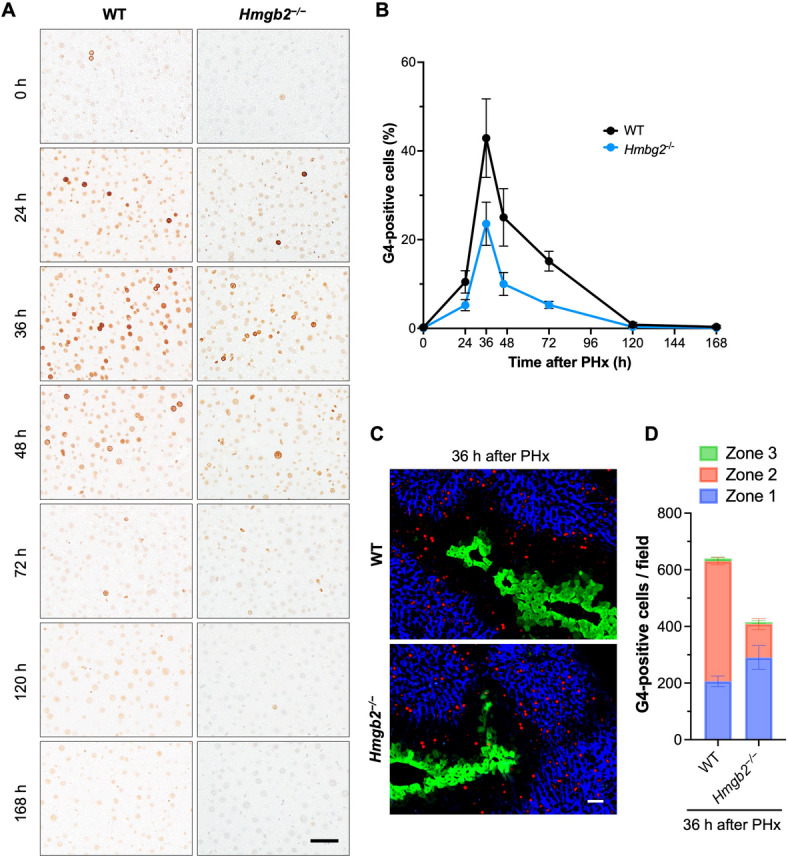
Loss of HMGB2 leads to reduced G4 formation during liver regeneration. (**A**) Immunohistochemical staining of G4 in the liver of WT and *Hmgb2*^*−/−*^ mice post-PHx. Scale bar, 50 μm. (**B**) Quantification of G4-positive hepatocytes in the liver of WT and *Hmgb2*^*−/−*^ mice post-PHx. Data represent the mean ± SEM from 5–6 mice per group. (**C**) Immunofluorescence analysis of G4 (red), E-cad (blue), and GS (green) expression in the liver of WT and *Hmgb2*^*−/−*^ mice at 36 h post-PHx. Scale bar, 100 μm.

A previous chemical profiling study using G4-binding small molecules identified HMGB2 as a potential G4-binding protein^42^. To determine the dynamics of G4 during liver regeneration in HMGB2-deficient mice (Supplementary Fig. S5), we examined G4 localization in the liver of *Hmgb2*^*−/−*^ mice post-PHx. In WT mice, > 40% of hepatocytes were G4 positive. By contrast, the proportion of G4-positive cells in regenerating liver of *Hmgb2*^*−/−*^ mice was less than half that observed in WT mice (Fig. 5A). Nevertheless, the temporal dynamics of G4 formation in *Hmgb2*^*−/−*^ mice exhibited a pattern comparable to that of WT mice, with G4 formation beginning 24 h post-PHx, peaking at 36 h, then subsequently declining (Fig. 5B). These results suggest that the G4 stability depends on the presence of specific proteins that promote or stabilize the structure.

Interestingly, in addition to a significant reduction in the overall level of G4 formation in *Hmgb2*^*−/−*^ mice, G4-positive hepatocytes within liver lobules were spatially distributed not only in zone 2 (midzonal) but also abundantly in zone 1 (periportal) at 36 h post-PHx (Fig. 5C). Quantification and comparison of the number of G4-positive cells in each hepatic zone at 36 h post-PHx (when G4 formation peaks) showed that in WT mice, zone 1 accounted for 32.2 ± 1.7% of all G4-positive cells, whereas zone 2 accounted for 66.3 ± 1.1%. By contrast, in *Hmgb2*^*−/−*^ mice, zone 1 accounted for 68.4 ± 5.7% of all G4-positive cells, and zone 2 accounted for 21.9 ± 2.1%, indicating a substantial shift in the zonal distribution of G4 formation (Fig. 5D). These findings highlight the critical role of HMGB2 as a determinant of G4 stability in regenerating liver, linking the chromatin architecture to the regulation of secondary DNA structure dynamics. These data validate the use of G4-binding protein KO mice as an in vivo model for assessing the roles of specific proteins in G4 DNA formation.

## Discussion

In this study, we provide the first comprehensive characterization of the in vivo dynamics of DNA G4 formation during liver regeneration following PHx in mice. Using a G4-specific antibody for immunohistochemistry analysis, we visualized the spatiotemporal distribution of G4 structures in regenerating hepatocytes and correlated G4 formation with the cell cycle phase and zonation within hepatic lobules. Using *Hmgb2*^*−/−*^ mice, we also assessed the role of a candidate G4-associated protein in regulating G4 formation in vivo. Collectively, our findings underscore a previously uncharacterized dimension of G4 biology in regenerating tissues and provide a platform for further exploration of G4-mediated genome regulation in physiological contexts.

Our immunohistochemical analyses demonstrated that the number of G4-positive hepatocytes increases rapidly post-PHx, peaking at 36 h before declining as liver mass is restored. This temporal pattern closely coincides with the onset of DNA synthesis during hepatocyte proliferation, suggesting that G4 formation is tightly linked to S-phase progression. Indeed, in analyses of colocalization of G4 with cell cycle markers, including EdU, cyclin A2, and PCNA, G4 formation was most prominent in S-phase hepatocytes, whereas minimal colocalization was observed in quiescent (G_0_/G_1_) or mitotic (M-phase) cells. The results of super-resolution imaging further support phase-specific behavior, as they showed that G4s localize in a dot-like pattern within nuclei during early S phase but exhibit a more diffuse distribution in late S phase. These observations are consistent with those of previous in vitro studies indicating that G4s form transiently during DNA replication^58,73^. Our in vivo data extend these findings by demonstrating that G4 formation in a complex tissue context is both temporally orchestrated and functionally linked to the cell cycle. Notably, we observed that G4 formation during liver regeneration exhibits clear zonation within the hepatic lobule. The peak in the number of G4-positive hepatocytes was observed predominantly in zone 2 (midzonal), particularly in the marginal zone adjacent to zone 1 (periportal), with lower numbers detected in zone 1 and only a few detected in zone 3 (pericentral). By 48–72 h post-PHx, the distribution of G4-positive hepatocytes shifts as they spread into zone 1 and later appear in zone 3. This dynamic zonal redistribution is intriguing, as it is known that hepatocytes are not uniform and are influenced by metabolic and microenvironmental heterogeneity intrinsic to the liver lobule^59,74^. Consistent with this, G4 formation was observed to coincide with the proliferative capacity of the lobular microenvironment. In addition, midzonal hepatocytes are known to exhibit high plasticity and proliferative potential in response to regenerative stimuli, and the preferential formation of G4s in zone 2 likely reflects this region-specific proliferative capacity. Such spatial distribution pattern of G4 formation suggests that it is modulated not only by intrinsic cell cycle cues but also by extrinsic factors, including oxygen tension, nutrient availability, and paracrine signaling within the liver microenvironment.

The colocalization of G4 signals with markers of DNA synthesis, but not with cyclin D1 or the mitotic marker pH3S10, supports the hypothesis that G4s function predominantly in the S phase of the cell cycle. The observed peak in G4 formation at 36 h post-PHx aligns with the early S phase, a critical window in which hepatocytes initiate DNA synthesis to compensate for lost tissue mass^75^. This temporal correlation underscores the potential role of G4s as modulators of genome replication fidelity and cell cycle progression in a regenerative context. Interestingly, the dot-like nuclear pattern observed in early S-phase hepatocytes may represent discrete G4 clusters at replication origins or regulatory loci. In contrast, the diffuse signals surrounding these dot-like foci observed in late S phase may reflect the resolution or remodeling of G4 structures as DNA synthesis progresses. This dynamic behavior aligns with in vitro observations indicating that helicases and other G4-binding proteins can stabilize or resolve G4s^31^, suggesting that G4 formation in vivo is reversible and regulated. Such transient stabilization may prevent “unscheduled” replication stress, thereby preserving genome integrity during rapid tissue regeneration.

To dissect the contribution of specific nuclear proteins to G4 dynamics, we examined liver regeneration in *Hmgb2*^*−/−*^ mice. Our findings revealed a striking reduction in the overall number of G4-positive hepatocytes compared with WT controls, although the temporal peak at 36 h post-PHx was preserved. This indicates that HMGB2 is not essential for the timing of G4 formation but is critical for stabilizing these structures and possibly enhancing the efficiency of their formation. Moreover, the spatial distribution of G4s within the lobule was altered in *Hmgb2*^*−/−*^ mice, with signals appearing in both zones 1 and 2, suggesting that HMGB2 contributes to the zonal regulation of G4 formation. These observations establish HMGB2 as a key determinant of G4 stability in vivo and highlight its role in coordinating chromatin architecture with secondary DNA structure dynamics during liver regeneration. Importantly, our previous study demonstrated that HMGB2 deficiency leads to a significant delay in liver regeneration^62^, supporting the reduced G4 formation observed in the liver of *Hmgb2*^*−/−*^ mice in the present study. Comprehensive analyses, including assessment of DNA damage responses, replication stress markers, and regeneration kinetics, would further deepen our understanding of the functional contribution of G4 structures and therefore represent important directions for future research.

HMGB2 induces structural changes in DNA, including bending, kinking, and unwinding, thereby functioning as a mediator of DNA conformational dynamics^76^. HMGB2 binds DNA in a sequence-independent manner and exhibits high affinity for bent or distorted DNA, as well as semi-catenated DNA loops, thereby enhancing DNA flexibility^77–79^. Although no studies have reported that HMGB2 directly binds G4s, it has been observed near G4s^42^, suggesting a potential role in modulating their local environment, even though its precise function remains unclear. Together, these findings suggest that other factors, potentially including additional G4-binding proteins, may partially compensate for the loss of HMGB2, thereby preserving the overall timing of S-phase progression. However, the reduced abundance and altered distribution of G4s in *Hmgb2*^*−/−*^ mice likely have functional consequences that potentially affect the efficiency of DNA replication and the fidelity of transcriptional programs necessary for proper liver regeneration.

Previous studies have provided indirect evidence that G4s play physiological roles, largely obtained through chemical stabilization^35,80–83^ or disruption^84,85^ using cell culture models. Our in vivo findings extend these observations by demonstrating that G4s are dynamically regulated in a tissue context and that specific nuclear factors, such as HMGB2, affect their abundance and spatial organization. The clear association between G4s and S-phase hepatocytes underscores their important roles in DNA synthesis and cell cycle control, suggesting that targeting G4 dynamics may have therapeutic potential in regenerative medicine and the treatment of liver disease. In future studies, it will be important to directly demonstrate the functional contribution of G4 structures to liver regeneration by administering small molecules that stabilize or destabilize G4 structures in conjunction with partial hepatectomy. These approaches will be essential to establish a causal link between G4 dynamics and liver regenerative processes.

The zonal specificity of G4 formation also has important implications for liver biology. Hepatocytes in different zones exhibit distinct metabolic profiles and proliferative capacities, reflecting functional heterogeneity^59^. Zone 2 hepatocytes preferentially repopulate the liver during homeostasis and also participate in regeneration when either zone 1 or zone 3 is injured by exposure to hepatotoxins^86^. By preferentially forming in midzonal hepatocytes, G4 structures may function as integrative molecular hubs that couple cell cycle signals with metabolic and transcriptional programs, thereby orchestrating efficient and coordinated liver regeneration. Disruption of G4 stability, as observed in *Hmgb2*^*−/−*^ mice, could perturb this integration, potentially leading to impaired regenerative outcomes or increased susceptibility to genome instability. Furthermore, our findings support a model in which G4s are not static structural elements but dynamic regulators of genome function in vivo. The phase- and zone-specific patterns observed in regenerating liver suggest that G4 formation is affected by a combination of intrinsic DNA sequence features, chromatin context, cell cycle status, and nuclear proteins. This multidimensional regulatory mechanism may represent a general principle applicable to other proliferative tissues, including the intestine, hematopoietic system, as well as cancerous tissues^87^, in which G4 dynamics might coordinate replication, transcription, and genome stability.

In summary, our study provides the first comprehensive characterization of DNA G4 dynamics in vivo during liver regeneration. G4 formation is temporally coordinated from the S phase through the G2 phase and exhibits zonal specificity within the liver lobule, which is modulated by HMGB2, a factor that promotes G4 structure formation. These findings establish a genetic framework for dissecting G4 regulation in vivo and highlight the functional significance of G4s as regulators of cell cycle progression and regenerative processes. Our results not only enhance the fundamental understanding of DNA secondary structure biology but also suggest potential avenues for therapeutic manipulation of G4s in regenerative medicine and disease contexts.

## Materials and methods

### Reagents and Chemicals

DNase I, RNase A, paraformaldehyde (PFA), bovine serum albumin (BSA), tyramine hydrochloride, and Brij L23 were purchased from Merck (Darmstadt, Germany). Click-iT Cell Reaction Buffer kit, 4’,6-diamidino-2-phenylindole (DAPI), 5/6-carboxyfluorescein, and 5/6-carboxytetramethylrhodamine succinimidyl esters and Permount mounting medium were purchased from Thermo Fisher Scientific Inc. (Waltham, MA, USA). 5-Ethynyl-2’-deoxyuridine (EdU) was purchased from Tokyo Chemical Industry Co., Ltd. (Tokyo, Japan). 3,3’-Diaminobenzidine tetrahydrochloride (DAB) was purchased from DOJINDO. Mayer’s hematoxylin solution, eosin alcohol solution, and all other reagents used in this study were purchased from Fujifilm Wako Pure Chemical Corporation (Osaka, Japan).

### Mice

Eight-week-old male C57BL/6J WT mice (average initial weight, 23.1 ± 2.2 g) were purchased from SLC Inc. (Shizuoka, Japan). The generation of genomic HMGB2 knockout (KO) mice was described previously^62,88,89^. To establish a consistent genetic background, HMGB2 KO mice were backcrossed onto C57BL/6J WT mice for more than five generations and subsequently maintained through heterozygous breeding. All mice were housed in standard animal cages under specific pathogen-free (SPF) conditions, maintained on a 12-h light/dark cycle at 22 °C, with ad libitum access to food and water. Prior to experimental procedures, mice were acclimated for 1 week. Mice were euthanized by overdose of inhaled isoflurane using an anesthetic device, NARCOBIT-E and an anesthetic induction chamber (Nazme Co. Ltd., Tokyo, Japan), followed by cervical dislocation.

### Partial hepatectomy (PHx) and tissue preparation

Mice were anesthetized by inhalation of 1.5% isoflurane (0.5 L/min), and 70% PHx was performed as previously described^90–92^. Briefly, the left posterior, left anterior, and right anterior segments were individually isolated and resected *en bloc* with a single ligature, resulting in minimal bleeding. Particular care was taken to preserve the integrity of the hilar structures, including the portal vein, biliary tract, and gallbladder. The liver was carefully manipulated using cotton-tipped applicators. Following PHx, mice were euthanized at 0, 24, 36, 48, 72, 120, or 168 h, and tissues were collected. A portion of collected liver tissue was fixed overnight in 4% PFA in phosphate-buffered saline (PBS) at room temperature and subsequently embedded in paraffin using standard methods. All mice were injected intraperitoneally with 10 mg/kg of EdU 2 h prior to tissue collection^93^. Collected tissues were processed for immunohistochemistry and EdU staining. Four to seven mice were included in each experimental group. The number of mice was as follows: 4 in the untreated control group, 4 at 0 h after PHx, 4 at 24 h, 7 at 36 h, 5 at 48 h, 7 at 72 h, 5 at 120 h, and 6 at 168 h after PHx.

### Immunohistochemistry and immunofluorescence analyses

Paraffin-embedded tissues were cut into 5-µm-thick sections and placed onto silane-coated slide glasses (Matsunami Glass Ind., Ltd., Osaka, Japan). The sections were deparaffinized with toluene, rehydrated using a graded ethanol series, and then autoclaved at 120 °C for 15 min in 10 mM citrate buffer (pH 6.0). After inhibition of endogenous peroxidase activity by incubation with 3% hydrogen peroxide in methanol for 30 min (for DAB detection, the samples were treated with 0.3% hydrogen peroxide in methanol for 15 min), the sections were pre-incubated with 500 µg/mL normal goat IgG or rabbit IgG in 1% BSA in PBS for 1 h to block non-specific binding of antibodies. The sections were then reacted with the following primary antibodies for 16–17 h: anti–DNA G-quadruplex (G4), clone 1H6 (cat no. MABE1126, Merck, 1:200 for immunohistochemistry, 1:3000 for immunofluorescence), anti–E-cadherin (cat no. 610182, BD Biosciences, 1:100), anti–glutamine synthetase (cat no. 11037-2-AP, Proteintech, 1:200), anti–Ki-67 (cat no. M7248, Dako, 1:25), anti–Cyclin D1 (cat no. 2978, Cell Signaling Technology, 1:1000), anti–proliferating cell nuclear antigen (PCNA) (cat no. M0879, Dako, 1:2000), anti–Cyclin A2 (cat no. ab181591, Abcam, 1:2000), and anti–phosphorylated H3S10 (cat no. 9708, Cell Signaling Technology, 1:400). After washing with 0.075% Brij L23 in PBS, the sections were reacted with horseradish peroxidase (HRP)-conjugated goat anti-mouse IgG or HRP-conjugated goat anti-rabbit IgG for 1 h. After washing in 0.075% Brij L23 in PBS, the HRP sites were visualized using fluorescein isothiocyanate–conjugated tyramide and then microwaved at 95 °C for 15 min in 10 mM citrate buffer (pH 6.0). Samples were then reacted with the next primary antibody overnight, and detection was repeated with rhodamine-conjugated tyramide, followed by DAPI counterstaining^94,95^. Finally, the sections were mounted using Fluoromount.

For DAB detection, nuclei were stained with Mayer’s hematoxylin solution, and the sections were then mounted using Permount mounting medium. In some experiments, glutamine synthetase was visualized using Alexa Fluor 633–conjugated goat anti-rabbit IgG. As a negative control for each experiment, normal mouse, rabbit, or goat IgG was used at the same concentration instead of the primary antibody. EdU was detected using a Click-iT Cell Reaction Buffer kit according to the manufacturer’s instructions. To verify the specificity of the G4 antibody, consecutive sections were pretreated with DNase or RNase before antibody labeling. Specifically, the sections were incubated with either 0, 25, or 250 µg/mL DNase I or 0, 10, or 100 µg/mL RNase A for 1 h at 37 °C. For neutralization assays, the G4-forming DNA oligonucleotide d(TTTTGGGG)_2_ was heated at 95 °C for 5 min and then slowly cooled to room temperature to allow the formation of G4 structures in the presence of 1 M NaCl, 10 mM Tris-HCl (pH 7.5), and 1 mM EDTA (final concentration of oligonucleotide: 100 µM). An excess amount of G4-structured DNA oligonucleotide (100×) was mixed with the antibody and incubated at room temperature for 1 h, after which the mixture was applied to the liver tissue sections. Images were captured using a BX53 Biological Microscope (Olympus) with a DP74 Microscope Digital Camera (Olympus), FV4000 confocal laser scanning microscope (EVIDENT), SpinSR10 Super-Resolution Imaging System (EVIDENT), and All-in-One Fluorescence Microscope BZ-X810 (Keyence) with DAPI-V (*λ*_ex_ 395/25 nm, *λ*_em_ 460/50 nm), GFP (*λ*_ex_ 470/40 nm, *λ*_em_ 525/50 nm), TRITC (*λ*_ex_ 545/25 nm, *λ*_em_ 605/70 nm), and Cy5 (*λ*_ex_ 620/60 nm, *λ*_em_ 700/75 nm) filters. cellSens imaging software (version 3.1, Olympus) was used for image acquisition and analysis.

### Cell culture and immunocytochemistry analysis

HepG2 (hepatocellular carcinoma) were cultured in Dulbecco’s modified Eagle’s medium (DMEM) supplemented with L-glutamine and phenol red and 10% heat-inactivated fetal bovine serum (FBS) and penicillin-streptomycin-amphotericin B at 37 °C with 5% CO_2_. For cell synchronization, HepG2 cells were incubated for 24 h in serum-free DMEM (for G_0_/G_1_) and for 3 h in DMEM with 10% FBS (for S phase). The cells were seeded at a density of 5 × 10^4^ cells per well in 24-well plates and subjected to immunocytochemical analysis. Cells grown on glass coverslips (Matsunami Glass Ind., Ltd.) were fixed in 4% PFA in PBS for 15 min and permeabilized using 0.2% Triton X-100 in PBS for 10 min at room temperature. After blocking with 500 µg/mL normal goat IgG in 1% BSA in PBS for 1 h, immunocytochemistry analysis was performed using standard methods with anti–DNA G4, clone 1H6 and Alexa Fluor 546–labeled goat anti-mouse IgG (A11003, Invitrogen) antibodies. Cell nuclei were counterstained with DAPI. Coverslips were mounted using Fluoromount.

### Quantitative analysis

Immunohistochemical staining was quantitatively analyzed using ImageJ software (NIH, Bethesda, MD, USA). Digital images were acquired under identical microscope settings. After color deconvolution using the H DAB vector, the DAB channel was extracted and converted to grayscale. In ImageJ, pixel intensity values range from 0 to 255, where 0 represents the darkest shade and 255 represents the lightest shade. Based on these values, staining intensities were classified into four categories: negative, weak, moderate, and strong. Specifically, values of 0–100 were defined as strong, 101–160 as moderate, 161–200 as weak, and values above 200 as negative. In this study, only cells exhibiting strong staining were considered G4-positive. Cells were quantified in 10 randomly selected fields (5 pericentral and 5 periportal regions) per section at ×200 magnification (approximately 2,000 hepatocytes). For each liver section, cells positive for G4, Ki-67, Cyclin D1, EdU, Cyclin A2, pH3S10, and PCNA in remnant liver lobes were counted in 10 randomly selected areas at ×200 magnification, as described above.

### Statistics and reproducibility

All data are presented as the mean ± standard error of the mean (SEM). Statistical analyses and generation of graphs and plots were performed using GraphPad Prism 10 (GraphPad Software, San Diego, CA, USA). Statistical comparisons of data sets were performed using one-way analysis of variance followed by Dunnett’s multiple comparisons test (for Fig. 1B and D) or using a mixed-effects model followed by Tukey’s multiple comparisons test (for Fig. 4C), with significance thresholds of *p < 0.05, **p < 0.01, or ***p < 0.001. Differences were considered significant at *p* < 0.05. All experiments were independently repeated at least three times, with reproducible results.

## Supplementary Information

Below is the link to the electronic supplementary material.

Supplementary Material 1

## Data Availability

All data supporting the findings of this study are included in the main article and its Supplementary Materials.
