# Supplementary material for: In vivo dynamics of G-quadruplex DNA structures during liver regeneration in mice
Source: Sci Rep. 2026 Apr 28;16:19751. doi: 10.1038/s41598-026-51144-3 (PMC13316107; doi:10.1038/s41598-026-51144-3)
Supplement: Supplementary file 1 — Supplementary Material 1 [file 41598_2026_51144_MOESM1_ESM.pdf]

Supplementary information

In vivo dynamics of G-quadruplex DNA structures during liver regeneration in mice

Takumi Ishizuka[1,†,*], Kham Mo Aung[1,†], Baljinnyam Lkham-Erdene[1,2], Koichi Yano[1,3], Toshiki Kubota[1,4], Fidya[1,5], Shinichiro Shirouzu[1,4], Makoto Ikenoue[1,3], Kengo Kai[1,3], Phyu Synn Oo[1], Narantsog Choijookhuu[1,6] and Yoshitaka Hishikawa[1,7,*]

[1] Department of Anatomy, Histochemistry and Cell Biology, Faculty of Medicine, University of Miyazaki, 5200 Kihara, Kiyotake, Miyazaki 889-1692, Japan
[2] Department of Thoracic Surgery, National Cancer Center of Mongolia, Nam Yan Ju street, Bayanzurkh District, Ulaanbaatar 13370, Mongolia
[3] Department of Surgery, Faculty of Medicine, University of Miyazaki, Miyazaki 889-1692, Japan
[4] Department of Oral and Maxillofacial Surgery, Faculty of Medicine, University of Miyazaki, 5200 Kihara, Kiyotake, Miyazaki 889-1692, Japan
[5] Department of Oral Biology, Faculty of Dentistry, Universitas Brawijaya, Malang, Jawa Timur, 65145, Indonesia
[6] Present Address: Department of Pathology and Forensic Medicine, School of Biomedicine, Mongolian National University of Medical Sciences, Zorig street, Ulaanbaatar 14120, Mongolia
[7] Frontier Science Research Center, University of Miyazaki, 5200 Kihara, Kiyotake, Miyazaki 889-1692, Japan

* To whom correspondence should be addressed. Email: tishizuka@med.miyazaki-u.ac.jp, yhishi@med.miyazaki-u.ac.jp

[†] These authors contributed equally

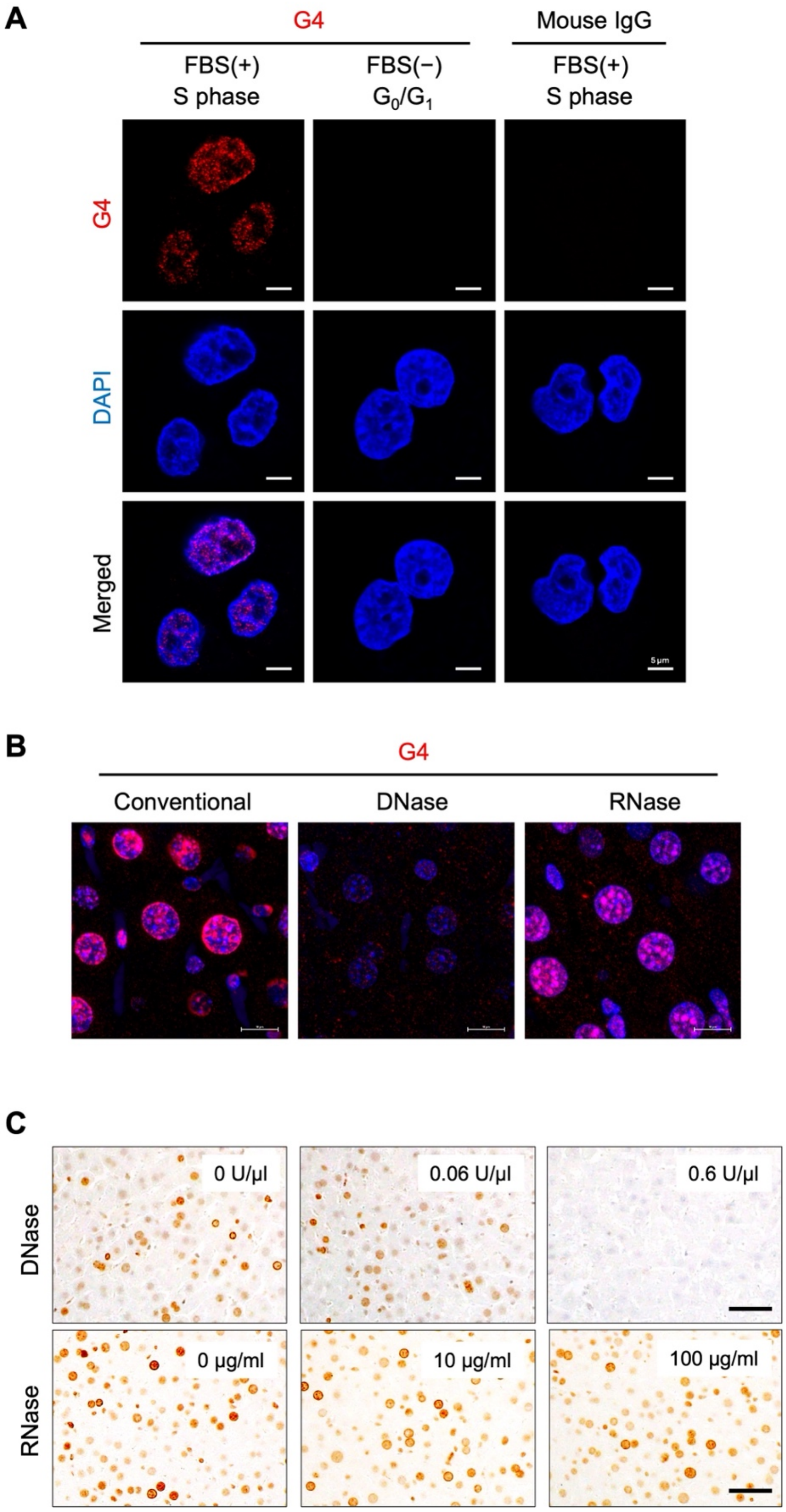

Supplementary Figure 1 Dynamic changes of G4 structures during cell cycle progression. (A) G4-specific antibody (1H6) staining of synchronized HepG2 cell populations at G_0/G_1 phase and during the S phase. Nuclei are counterstained with DAPI (blue). Scale bars, 5 μm. (B) Control assays for 1H6 immunoreactivity in liver tissue 36 h after PHx. The liver tissue was treated with DNase and RNase before incubation with the anti-G4 antibody. Nuclei were counterstained with DAPI. Scale bar, 10 μm. (C) Control assays for anti-G4 antibody immunoreactivity in liver tissues. Liver tissues at 36 h post-PHx were treated with DNase (upper) and RNase (bottom) before incubation with the anti-G4 antibody. Nuclei were counterstained with hematoxylin. Scale bar, 50 μm.

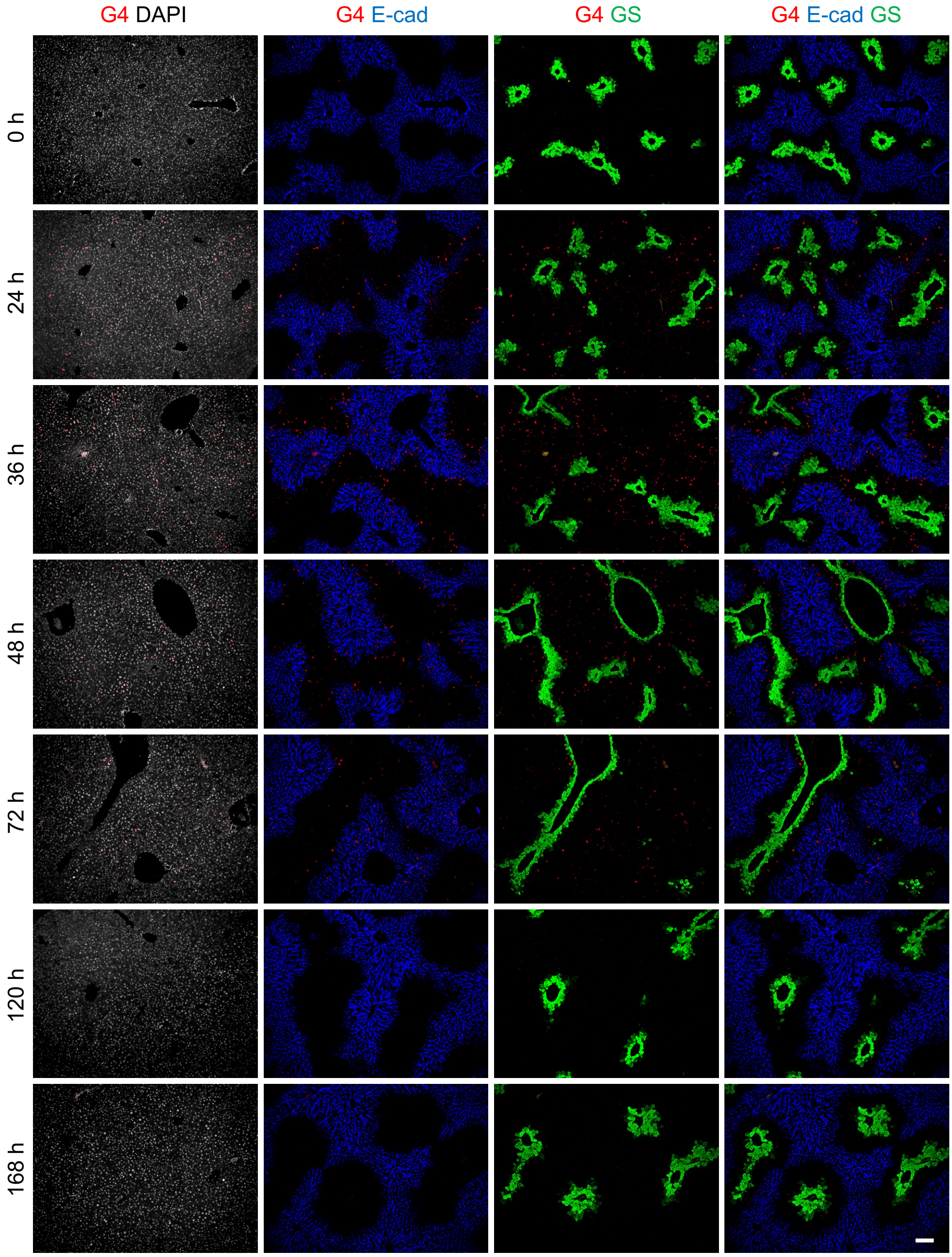

Supplementary Figure 2 Immunofluorescence of G4 (red), E-cad (blue), GS (green), and DAPI (white) in the liver of mice post-PHx. Time after PHx is shown on the left. Scale bar, 100 μm.

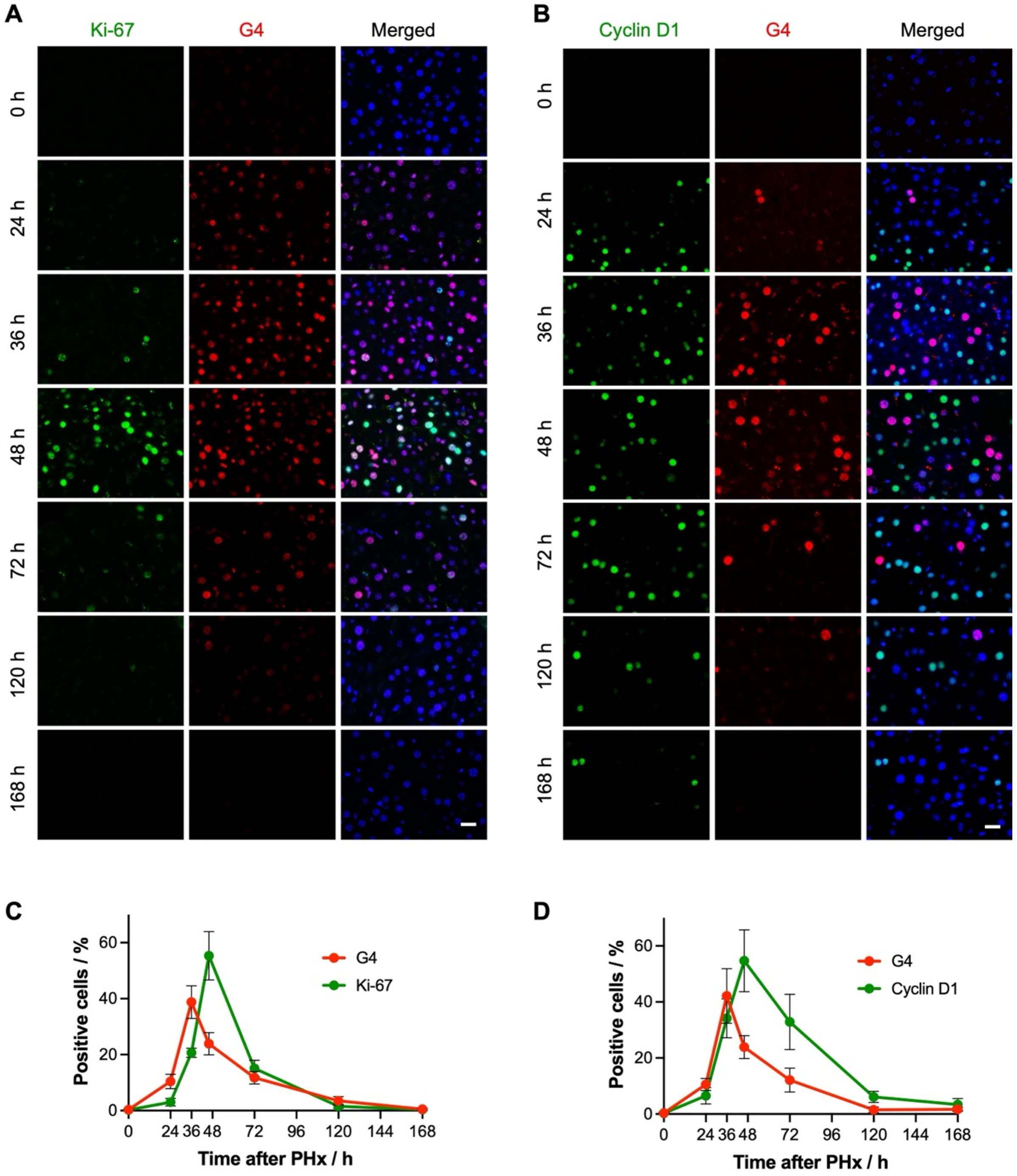

Supplementary Figure 3 Colocalization of G4 with Ki-67 and cyclin D1 during liver regeneration. (A) Immunofluorescence of Ki-67 (green) and G4 (red) in the liver of mice post-PHx. (B) Immunofluorescence of cyclin D1 (green) and G4 (red) in the liver of mice post-PHx. Scale bar, 20 μm. Number of Ki-67–positive (C) and cyclin D1–positive (D) cells in the liver of mice post-PHx. Data represent the mean ± SEM from 4-7 mice per group.

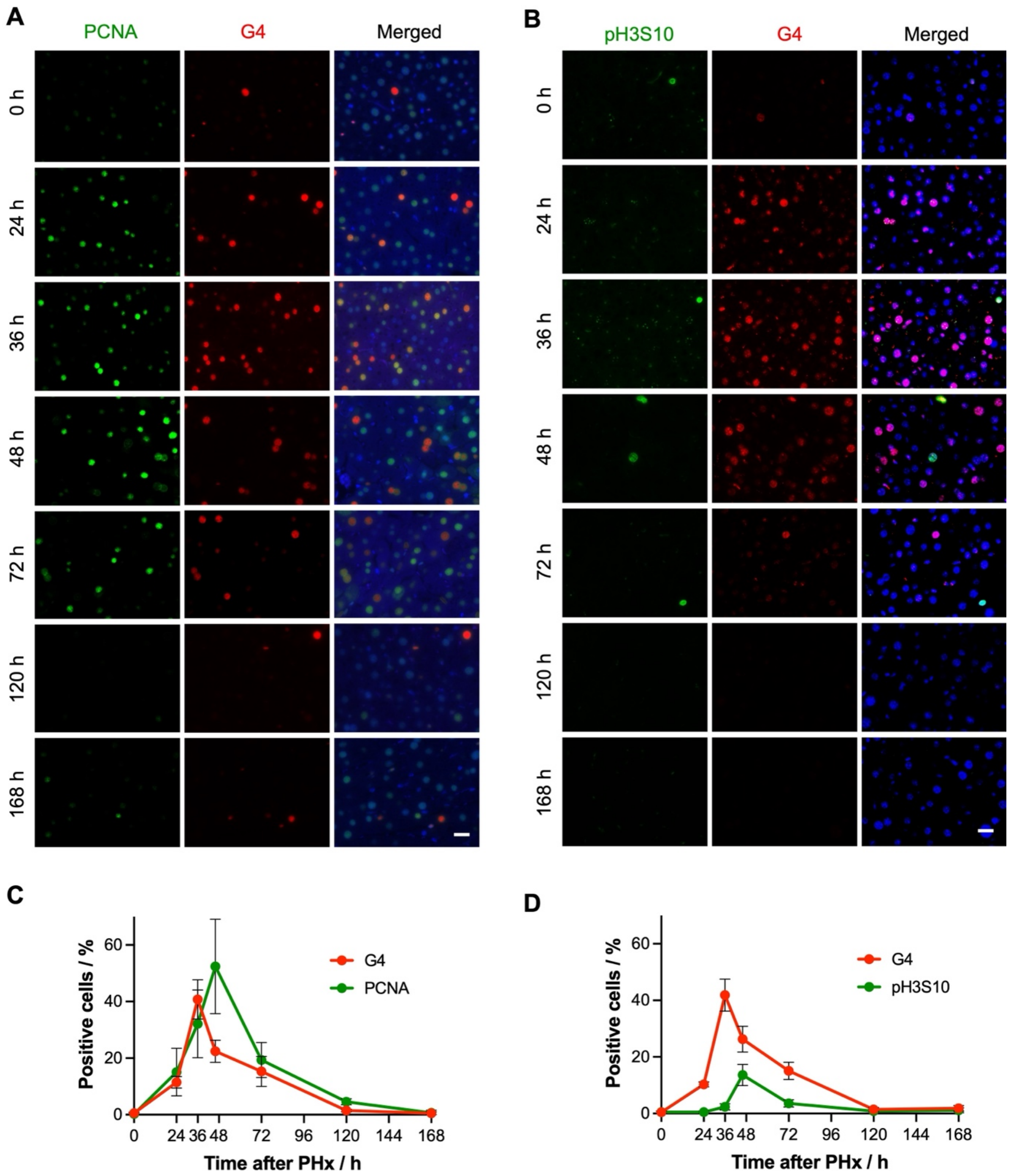

Supplementary Figure 4 Colocalization of G4 with PCNA and pH3S10 during liver regeneration. (A) Immunofluorescence of PCNA (green) and G4 (red) in the liver of mice post-PHx. (B) Immunofluorescence of pH3S10 (green) and G4 (red) in the liver of mice post-PHx. Scale bar, 20 μm. Number of PCNA-positive (C) and pH3S10-positive (D) cells in the liver of mice post-PHx. Data represent the mean ± SEM from 4-7 mice per group.

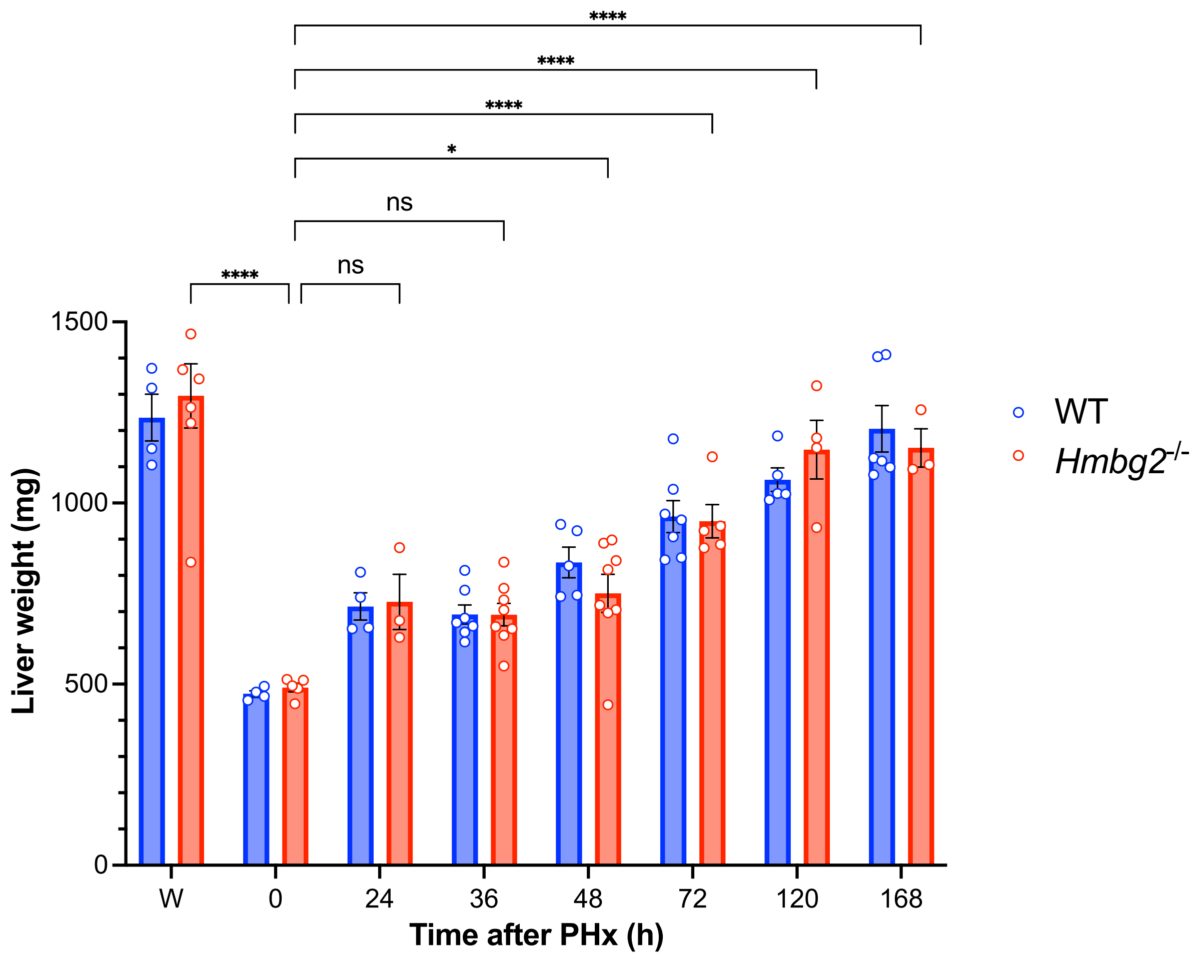

Supplementary Figure 5 Change in liver weight during regeneration in WT and *Hmgb2*$^{-/-}$ mice post-PHx. Data represent the mean ± SEM for 4-8 mice. The *p* values for differences between each time point and day 0 were calculated using one-way ANOVA followed by Dunnett's test (*p< 0.05, **p<0.01, ***p<0.001). The WT dataset (blue) is the same as that shown in Figure 1B.
